# Propranolol pharmacokinetics in infants treated for Infantile Hemangiomas requiring systemic therapy: Modeling and dosing regimen recommendations

**DOI:** 10.1002/prp2.399

**Published:** 2018-04-30

**Authors:** Laurence Del Frari, Christine Léauté‐Labrèze, Laurent Guibaud, Sébastien Barbarot, Jean‐Philippe Lacour, Christine Chaumont, Alain Delarue, Jean‐Jacques Voisard, Valérie Brunner

**Affiliations:** ^1^ PKPD Department Pierre Fabre Médicament Toulouse France; ^2^ Unité de Dermatologie Pédiatrique Hôpital Pellegrin‐Enfants Bordeaux France; ^3^ Consultation des angiomes Imagerie pédiatrique et fœtale Hôpital Mère –Enfant Lyon France; ^4^ Département Dermatologie Hôtel‐Dieu Nantes France; ^5^ Département Dermatologie Hôpital L'Archet Nice France; ^6^ Development Department Pierre Fabre Dermatologie Toulouse France; ^7^ Medical Department Pierre Fabre Dermatologie Toulouse France; ^8^ Pierre Fabre Dermatologie Toulouse France; ^9^ SERVIER Laboratories Center of Excellence Pharmacokinetics Suresnes France

**Keywords:** Pharmacokinetic Population analysis Modeling and Simulation Dermatology Pediatrics

## Abstract

Propranolol has become the first choice therapy for complicated Infantile Hemangiomas (IH). The pharmacokinetics of propranolol were evaluated after repeated oral administration of a new pediatric solution of propranolol at 3 mg kg^−1^ day^−1^ given twice daily (BID) in infants (77‐243 days) with IH. A population model was built to describe the pharmacokinetics of propranolol in infants and to simulate different dosing regimens. One hundred and sixty‐seven plasma concentrations from 22 infants were used in the population analysis. Weight effect was tested on apparent clearance and volume of distribution. Monte‐Carlo simulations were performed for 4 dosing regimens: BID dosing with irregular or strict 12‐hour intervals and 2 different 3 time daily dosing (TID) regimens. The best model was a one‐compartment model with first‐order absorption and elimination rates. The weight affected the clearance but not the volume. Typical oral clearance was estimated at 3.06 L hour^−1^ kg^−1^ (95% CI: 1.14‐8.61 L hour^−1^ kg^−1^), close to adult clearance data. When regular BID dosing was compared to TID or irregular BID regimens, simulated median Cmin and Cmax were <20% different. To conclude, a model using a weight allometric function on clearance was established and confirmed that the dose in mg/kg should be used without adaptation by range of age in treatment of complicated IH. The simulations support the use of a BID dosing preferably to a TID dosing thanks to close Cmin and Cmax at steady state between both regimen and showed the possibility of irregular BID dosing, allowing early administration in the evening when needed.

**Abbreviations**
AIC: Akaike Criterion
BID: *bid in die* Twice a day
BSV: between subject variability
CL/F: total apparent plasma clearance
CWRES: conditional‐weighted residuals
GOF: goodness of fit
IH: infantile Hemangiomas
IPRED: individual predicitions
IWRES: individual‐weighted residuals
OFV: objective function value
Pc VPC: prediction corrected VPC
PRED: population prediction
V/F: apparent volume of distribution
VPC: visual predictive check

## 1 INTRODUCTION

Hemangiomas affect about 4% of all infants, and up to 30% of premature babies. Although 85%‐90% of all infantile hemangiomas (IH) eventually undergo spontaneous involution, they can cause disfigurement and serious complications depending on their location (obstruction of airways and vision), size (cardiac insufficiency, hypothyroidism), and speed of regression. They can be associated with painful ulceration and hemorrhage. Treatment options for complicated hemangiomas include oral steroids, laser, surgery, cryotherapy, and vincristine, interferon or cyclophosphamide for life‐threatening cases. Each of these options has its restrictions and⁄or related side effects.

Since 2008,^22^ propranolol has become the first choice therapy for complicated IH.^35^ The current knowledge of mechanism of action of propranolol on IH evokes 3 possible concomittant mechanisms: vasoconstriction of the high‐flow blood vessels feeding the IH tumor, VEGF growth factor suppression, and downregulation of other proangiogenic cytokines^15^, ^35^

Propranolol hydrochloride is a nonselective beta‐adrenergic blocking agent. It has been in clinical use since the 1960's, and is commonly prescribed worldwide for cardiovascular diseases. In children, specific dosing recommendations have been established and its clinical use is accepted in hypertension, arrhythmias, tetralogy of Fallot spells, hypertrophic myocardiopathy, and thyrotoxicosis.

In adults, propranolol is almost completely absorbed after oral administration.^31^ It undergoes a high first‐pass metabolism by the liver and on average <30% of propranolol reaches the systemic circulation.^1^, ^8^, ^9^ Maximum plasma drug concentrations (*C*_max_) occur approximately 1‐2 hours after an oral dose.^9^, ^31^, ^36^, ^41^ Approximately 90% of circulating propranolol is bound to plasma proteins.^12^ Its volume of distribution is approximately 4 L kg^−1^.^9^, ^31^ Propranolol is extensively metabolized through three primary routes: ring hydroxylation, side‐chain oxidation, and direct glucuronidation involving mainly CYP2D6, CYP1A2, and UGT enzymes, respectively. Its half‐life ranges from 3 to 6 hours.^8^, ^9^, ^36^, ^41^ Propranolol is excreted as metabolites in urine, <1% of a dose being excreted as unchanged drug in the urine.^39^

In infants, limited information was available on the pharmacokinetic profile and plasma exposure of propranolol after oral administration. Until 2013, only 92 plasma concentrations observed after oral administration of propranolol in children from 8 weeks‐ to 13 years‐old were documented in 3 publications: mean concentrations ranged between 0.05 and 57 ng mL^−1^ for different doses and regimen.^32^, ^34^, ^40^ In 2013,^14^ published results in 4 term and 23 preterm neonates treated with oral propranolol at 0.25 or 0.5 mg kg^−1^ every 6 hour in which 1000 concentrations were measured by serial dried blood spots. After 0.5 mg/kg/6 hour, the mean maximum concentrations were 71.7 ± 29.8 ng mL^−1^.

A new pediatric oral solution of propranolol has been developed for the treatment of proliferative IHs requiring systemic therapy. Its efficacy has been demonstrated through one phase 2/3 dose ranging study at doses of 1 and 3 mg kg^−1^ day^−1^ with twice daily (BID) administration.^23^ A pharmacokinetic study was also performed in the same infant population, in which plasma concentrations of propranolol were measured during the titration period and at steady‐state at the target dose of 3 mg kg^−1^ day^−1^ given BID. Using these data, a population pharmacokinetic model was developed to describe the pharmacokinetics of propranolol in infants, to evaluate the between subjects variability (BSV) and as far as possible, to understand the source of the variability in this population. Finally, the population model was used to simulate different dosing regimens for supporting the administration of the propranolol solution in infants with IH.

## 2 MATERIALS AND METHODS

### 2.1 Study design

Propranolol plasma data were obtained from an open label, repeated dose study conducted in 4 hospitals in France. The clinical study was performed in accordance with the principles stated in the 1964 Helsinki declaration and its subsequent amendments, Good Clinical Practices (GCP; CPMP/ICH/135‐95) for trials on medicinal products and with Huriet Law of 20 December 1988, relating to the protection of individuals involved in biomedical research in France and its subsequent amendments. Approval was obtained by the Comité de Protection des Personnes of Sud Ouest et Outre Mer III, EudraCT number: 2009‐018102‐22. Informed written consent was obtained from all parents of participants included in the study.

No statistical determination of sample size was performed. The study was designed to characterize the pharmacokinetics (PK) of propranolol at steady‐state in infants administered with the pediatric oral solution. Infants were stratified to 2 groups according to their age at inclusion, which defined the timing of their PK assessment at steady‐state:

1. *Group 1*: infants aged from 35 to 90 days inclusive at inclusion; PK assessment after 4 weeks of treatment,
2. *Group 2*: infants aged from 91 to 150 days inclusive at inclusion; PK assessment after 12 weeks of treatment.

This stratification ensured to collect evaluable propranolol concentrations within the largest range of ages in a small group of infants. Twenty‐three infants were enrolled from May 28th, 2010 to June 7th, 2011. The main criteria for inclusion were as follows: age at inclusion from 35 to 150 days old inclusive, and presence of proliferating IH requiring systemic therapy. Treatment was initiated with a titration period: the initial dose was 1 mg kg^−1^ day^−1^ for 1 week, then the dose increased to 2 mg kg^−1^ day^−1^ during the second week to achieve the target dose of 3 mg kg^−1^ day^−1^ for 10 weeks. Treatment was given BID and was divided in 2 equal doses.

The dosing schedule was adapted to calculate an appropriate 12 hours exposure for the noncompartmental analysis (not shown) at the target therapeutic dose: in order to have a regular dosing time interval for the day of PK evaluation, it was requested to the parents that the evening administration before the day of full PK evaluation (D28 or D84) should be around 20:00 (last morning administration around 8:00) instead of around 17:00 for the other days. Available literature data did not allow to optimize the study design for the modeling approach.

### 2.2 Pharmacokinetic assessments

A total of 8 samples (250 μL each) per infant was collected during the study (Table 1). The quantification of propranolol was performed using a liquid chromatography with tandem mass spectrometry analytical method validated according to the Guidance for Industry: Bioanalytical Method Validation – FDA – May 2001. The quantification range of the method was 0.50 to 250 ng mL^−1^. Assay precision for propranolol in plasma samples ranged from 1.6% to 5.5%, with an accuracy of 97.4% to 101.6%.

**Table 1:** Sampling design

Day	Dose	Sampling time
D7	1 mg kg^−1^ day^−1^	Pre‐dose
D14	2 mg kg^−1^day^−1^	Pre‐dose
D28 (Group 1) or D84 (Group 2)	3 mg^−1^kg^−1^day	Pre‐dose T1 hour T2 hour T4 hour T6 hour T9 hour (before the afternoon dosing)

### 2.3 Pharmacokinetic modeling

#### 2.3.1 Base model and covariate model

One‐ and two‐compartment models were explored. In the one‐compartment model, absorption was investigated using a zero‐order and a first‐order absorption rate. The distribution and elimination processes were parameterized in terms of volumes and clearances. As the absolute bioavailability could not be determined using only oral plasma data, apparent parameters were estimated.

BSV was investigated on all parameters. The magnitude of BSV was estimated with an exponential error model (Equation 1), which implies a lognormal distribution of parameters. It was expressed, approximately, as a coefficient of variation (CV%):

(1) $$P_{j}=\text{TVP}∙\text{exp}\left(\eta_{j}\right)$$

where *P*_*j*_ is the parameter for the *j*th individual, TVP is the population parameter and η_*j*_ is the between‐subject random effect having mean 0 and variance to be estimated ω².

A proportional error model (Equation 2), or a combination of proportional error model and constant additive error model (Equation 3) were investigated to evaluate the residual error:

(2) $$C_{\mathrm{ij}}={\hat{C}}_{\mathrm{ij}}\cdot \left(1+\varepsilon_{1}\right)$$

(3) $$C_{\mathrm{ij}}={\hat{C}}_{\mathrm{ij}}\cdot \left(1+\varepsilon_{1}\right)+\varepsilon_{2}$$

where *C*_ij_ is the observed value, *Ĉ*_ij_ is the fitted value from the model, and ε_1_ and ε_2_ the random errors having mean 0 and variance to be estimated σ².

Body weight, age or groups of age, sex, dose, and time were the key covariates assessed during the clinical study. However, regarding the covariate selection, some characteristics of the study design have had an impact in the modeling approach:

1. *Stratification*: Infants were stratified in 2 groups according to their age at inclusion. Infants of Group 2 being older than those of Group 1, the mean weight at inclusion was higher (4.77 kg vs. 6.38 kg).
2. *Dose*: The target dose was expressed as mg/kg, and was individually calculated according to each infant weight.
3. *Timing of PK evaluation*: The PK evaluation was performed at steady‐state in both groups but after 4 weeks and 12 weeks of treatment for Group 1 and Group 2, respectively. At the PK evaluation time, the mean weight was 5.62 kg and 7.78 kg in Group 1 and Group 2, respectively.

**Figure 1:**
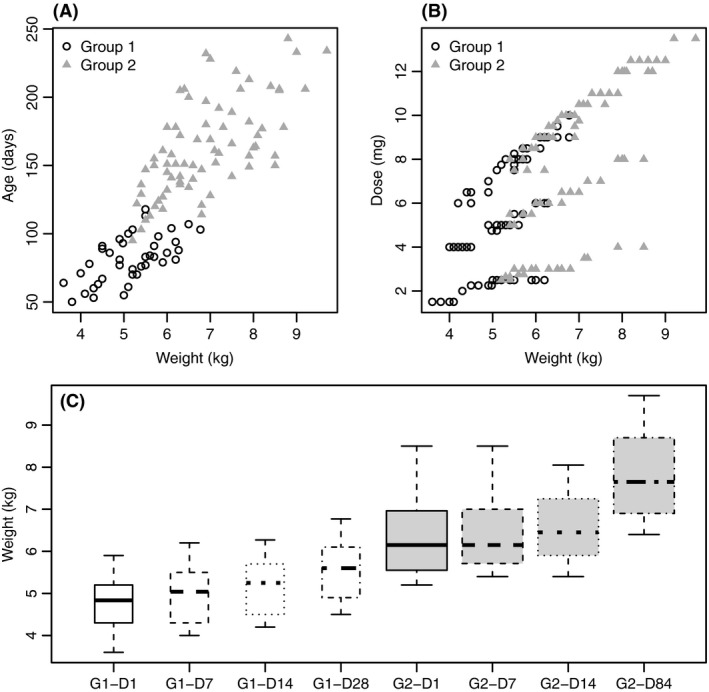
Covariate plots Age (days) versus weight (kg) (A) Dose (mg) versus weight (kg) (B) and Weight (kg) versus Day of visit (C). Empty Circles correspond to Group 1 and full triangles correspond to Group 2

Weight is the first covariate to be investigated in a pharmacokinetic modeling in infants in order to take into account the dose calculation in mg/kg and the growth and development of the infants.^17^, ^18^, ^19^, ^20^ As a consequence of the study design, the weight was highly correlated with the administered dose, the age and group of age, and the visit (time effect) (Figure 1) and was the only tested covariate.

The weight was evaluated on the clearance and the volume of distribution using allometric functions^4^, ^5^, ^17^, ^37^ (Equation 4):

(4) $${\text{TVP}}_{i}=P_{\mathrm{pop}}∙{\left(\frac{{\text{cov}}_{i}}{{\text{cov}}_{\mathrm{med}}}\right)}^{\theta }$$

where TVP_*i*_ represents the model‐predicted pharmacokinetic parameter (eg, total apparent plasma clearance CL/F) for the typical individual with covariate value cov_*i*_, *P*_pop_ represents the population central tendency for TVP, cov_med_ represents the median population value of the covariate, and θ represents the allometric coefficient.

### 2.4 Model selection criteria

The selection of the structural PK model was based on both NONMEM^®^ objective function value (OFV) and visual inspection of goodness of fit (GOF) plots. The difference in OFV between hierarchical models (likelihood ratio test) which is asymptomatically χ^2^ distributed, with a degree of freedom equal to the number of additional parameters of the full model compared to the reduced model, was used to declare superiority of one model to another (for nested models).

The evaluation of error models was performed using the GOF plots, the standard error of estimates and eta‐shrinkages. Correlations between BSV were estimated.

The weight effect was first examined on structural parameters by graphical assessment. Then, it was evaluated following a classical methodology for covariate inclusion^27^:

1. The weight covariate was added in an univariate manner on each structural parameter to determine if it significantly improves the model to the data. This was tested fixing or not the allometric coefficient to 0.75 and 1 for CL/F and V/F, respectively,
2. If the weight covariate was deemed significant based upon a change in OFV (ΔOFV) ≥ 3.84 (*P *< .05), for each structural parameter, it was retained for inclusion in a full model (ie, weight covariate effect on every structural parameters). Significance for fixing the allometric coefficient was tested based upon AIC criteria since the models were not nested.^28^
3. A backward deletion process was then performed where weight effect was deleted singly from the full model with the ΔOFV computed from the reduced and the full model. Weight effect that could be deleted from the full model without an associated increase in ΔOFV ≥ 6.64 (*P* < .01) were sorted by OFV and the covariate with the smallest ΔOFV removed from the model.

The first‐order conditional estimation method with η‐ε interaction option (FOCE INTER) was used to estimate the parameters of the model. For Goodness of Fit (GOF) Plots, population prediction calculated with first‐order estimation (PRED), conditional‐weighted residuals (CWRES), and individual‐weighted residuals (IWRES) were calculated.

### 2.5 Model qualification

GOF plots and standard errors of the final estimates were used to assess the fit to the observed data. Visual Predictive Checks (VPC) by visit and prediction corrected Visual Predictive Checks (pcVPCs) were also performed to evaluate the adequation of the model to the observed data. Monte‐Carlo simulations (1000 replications of the complete dataset) were performed and derived 5th, 50^th^, and 95th percentiles from the simulated data were superimposed to the observed concentrations and compared. Coverage plots for prediction intervals were provided.

### 2.6 Simulations of dosing regimens

The evaluation of propranolol pharmacokinetics was performed after fixing a regular 12 hour‐dosing interval in order to calculate an appropriate AUC_tau12 h_ for the noncompartmental analysis (not presented) and to decrease the unexplained variability in the modeling approach. On the contrary, in the phase 2/3 study,^23^ efficacy and safety of propranolol were evaluated in infants with BID repeated administrations without strictly fixing the administration timing. Due to possible irregular sleeping periods and meal times, dosing interval could be irregular.

Twice‐a‐day to four‐a‐day regimens for propranolol in proliferative IH are often described in the literature and in 2011, a consensus selected a TID administration.^11^, ^13^, ^16^, ^42^

Consequently, different administration schedules were simulated to compare the plasma profile obtained with the dosing regimens described above:

1. *First regimen*: BID administrations at clock times 8:00 am and 8:00 pm equal to regular 12‐hours intervals corresponding to our reference,
2. *Second regimen*: BID administrations at clock times of 8:00 am and 5:00 pm equal to a 9‐hour interval plus a 15‐hour interval (irregular dosing intervals over a 24‐hour period),
3. *Third regimen*: TID administrations at clock times 8:00 am, 14:00 am, and 8:00 am equal to two 6‐hour intervals and one 12‐hour interval over a 24‐hour period,
4. *Fourth regimen*: TID administration at clock times of 8:00 am, 12:00 am, and 8:00 pm equal to a 4‐hour interval followed by a 8‐hour interval and 12‐hour interval over a 24‐hour period.

Each schedule was repeated daily for 1 week at a total daily dose of 3 mg kg^−1^ day^−1^, divided in two administrations of 1.5 mg kg^−1^ or three administrations of 1 mg kg^−1^ for the BID and TID regimens, respectively.

The infant population of the study was replicated 1000 times by Monte‐Carlo simulations for each dosing regimen. The 5th, 50^th^, and 95th percentiles of the simulated concentrations were plotted versus time.

### 2.7 Softwares

Population PK analysis and Monte‐Carlo simulations were performed using NONMEM^®^ version 7.2 (Beal, NONMEM 7.2.0 users guides.^7^ Icon Development Solutions) on a personal computer with a GNU Fortran compiler and the interface PDx‐Pop version 5.1. SAS 9.3 (SAS Institute Inc^33^) was used for data management and descriptive statistics, R version 3.1.3 for GOF and simulation plots, PsN version 3.0^24^, ^25^ and Xpose version 4.4.1^6^, ^19^ for VPC graphics.

## 3 RESULTS

### 3.1 Demographics

Twenty‐three infants with proliferative IH (6 females, 16 males) were included in the study and 22 were evaluable for pharmacokinetic modeling. Among the 22 infants, 10 infants (3 females, 7 males) were included in the Group 1 and 12 infants (3 females, 9 males) in the Group 2. Descriptive statistics are given in Table 2.

**Table 2:** Summary demographics of infants at inclusion in the population pharmacokinetic analysis

Day	Group number	N	Mean age (days) (CV%)	Median age (days) (range)	Mean weight (kg) (CV%)	Median weight (kg) (range)
D1	Both Groups	22	103 (34.1%)	104 (50‐151)	5.6 (21%)	5.5 (3.6‐8.5)
	#1	10	70 (19.6%)	72 (50‐89)	4.8 (15%)	4.8 (3.6‐5.9)
	#2	12	131 (14.0%)	135 (95‐151)	6.4 (16%)	6.2 (5.2‐8.5)
D7	Both Groups	10	110 (32.3%)	112 (56‐159)	5.82 (19.4%)	5.71 (4.0‐8.5)
	#1	10	76 (18.4%)	79 (56‐96)	5.05 (15.0%)	5.04 (4.0‐6.2)
	#2	10	139 (13.0%)	141 (103‐159)	6.46 (15.3%)	6.15 (5.4‐8.5)
D14	Both Groups	12	118 (30.0%)	119 (63‐166)	5.98 (17.7%)	5.9 (4.2‐8.05)
	#1	12	84 (16.6%)	86 (63‐103)	5.25 (13.7%)	5.25 (4.2‐6.27)
	#2	12	146 (12.3%)	149 (110‐166)	6.59 (13.8%)	6.45 (5.4‐8.05)
D28	#1	10	98 (14.4%)	101 (77‐118)	5.62 (12.8%)	5.6 (4.5‐6.77)
D84	#2	12	216 (8.49%)	216 (180‐243)	7.78 (13.3%)	7.65 (6.4‐9.7)

### 3.2 Pharmacokinetic modeling

The analysis dataset included 167 observations (21 plasma concentrations at Day 7, 18 at Day 14, and 128 after administration of 3 mg kg^−1^ day^−1^ ‐ 60 in Group 1 and 68 in Group 2). Descriptive statistics of observed propranolol plasma concentrations are given in Table 3. Few data were missing: one concentration Below the Limit of Quantification (D14 Group 1), one sample (D7 Group 2), and one time information (D14 Group 2). One concentration (Day 84, Group 2, 1 hour‐post dose) equal to 448 ng mL^−1^ (confirmed by reanalysis) was considered as an outlier data. Initial model tests were performed including this concentration value. A graphical evaluation of the weighted residuals confirmed that the concentration was an outlier (+4.5 vs. [−2; +2]) leading to its exclusion from the dataset.

**Table 3:** Descriptive statistics of observed propranolol plasma concentrations (ng/mL)

Group Number	Day	Theoretical time (h)	n	Geometric mean (CV %)	Median (range)
1	D7	Predose	10	NA	4.05 (0.780‐12.5)
	D14	Predose	9	NA	8.46 (4.09‐15.8)
	D28	Predose	10	16.1 (75.5%)	17.5 (4.73‐36.8)
		1	10	62.9 (37.0%)	62.2 (28.0‐112)
		2	10	66.0 (36.7%)	61.8 (34.1‐119)
		4	10	47.4 (34.5%)	47.0 (24.0‐75.5)
		6	10	44.4 (42.9%)	37.2 (25.2‐87.3)
		9	10	31.7 (57.3%)	33.8 (13.7‐71.4)
2	D7	Predose	11	NA	3.69 (1.13‐29.9)
	D14	Predose	9	NA	5.86 (2.33‐25.5)
	D84	Predose	12	10.1 (113%)	9.74 (1.72‐47.4)
		1	10	37.7 (107%)	41.9 (13.4‐104)
		2	11	60.5 (79.9%)	74.3 (21.3‐125)
		4	12	40.3 (65.1%)	44.2 (12.2‐124)
		6	11	33.3 (69.4%)	31.4 (9.49‐112)
		9	12	23.4 (93.2%)	21.7 (5.22‐108)

NA, Not Applicable. Geometric mean and CV% were not calculated owing to a large range of sampling times.

### 3.3 Base model

The best structural model (or BASE model) describing propranolol pharmacokinetics was a one‐compartment disposition model with a first‐order absorption and a first‐order elimination. A two‐compartment was evaluated but it resulted in a significant increase in the OFV of + 106 units (*P *< .001) compared to the one‐compartment model. The residual error model was a proportional error model. BSV on all fixed parameters were estimated. The 95% confidence interval (CI) of the random effect relative to V/F included 0.

Parameter estimates are provided in Table 4. The 95% CI of each parameter did not contain zero except random effect of V/F. The relative standard errors (RSE generated from the NONMEM covariance step) of fixed parameter estimates were <30% and <50% for random parameter estimates indicating a good precision of estimates except for V/F.^28^, ^29^

**Table 4:** Propranolol base model: parameter estimates

Description of effects		Symbol	Estimate	95% CI (RSE %)	Variability (CV%)
Fixed effects	CL/F	θ_1_	19.9	15.7‐24.1 (10.8%)	
	V/F	θ_2_	137	112‐162 (9.20%)	
	Ka	θ_3_	1.08	0.484‐1.68 (28.1%)	
Random effects					
Between subjects’ variability	On CL/F	ω²_1_	0.238	0.0741‐0.402 (35.1%)	48.8
	On V/F	ω²_2_	0.0276	−0.0132 to 0.0684 (75.4%)	16.6
	On Ka	ω²_3_	2.40	0.185‐4.61 (47.1%)	155
Residual error	Proportional	σ²_1_	0.0974	0.0674‐0.127 (15.7%)	31.2

RSE, relative standard error of model parameter estimates obtained from the NONMEM covariance step; CI, confidence Interval calculated as estimate ± 1.96*RSE; CL/F, apparent total plasma clearance; V/F apparent volume of distribution; Ka, first‐order rate of absorption; θ; NONMEM fixed parameter; ω NONMEM between‐subject random effect; σ NONMEM residual random effect.

**Figure 2:**
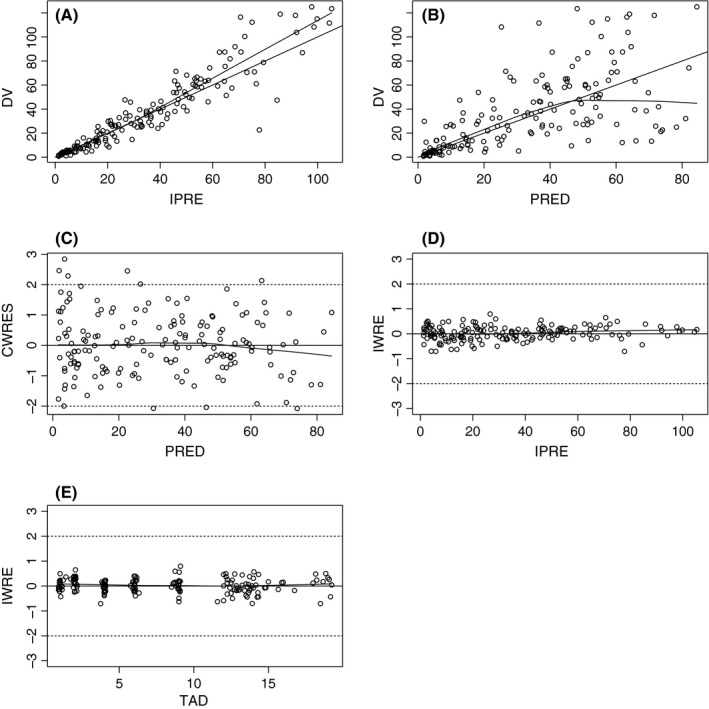
Base model goodness of fit plots Individual predictions (IPRED) versus observations (A) Population predictions (PRED) versus observations (B) conditional population‐weighted residuals (CWRES) versus population predictions (C) Individual predictions versus time after dose (H) (TAD) (D) and individual predictions versus individual‐weighted residuals (E)

**Figure 3:**
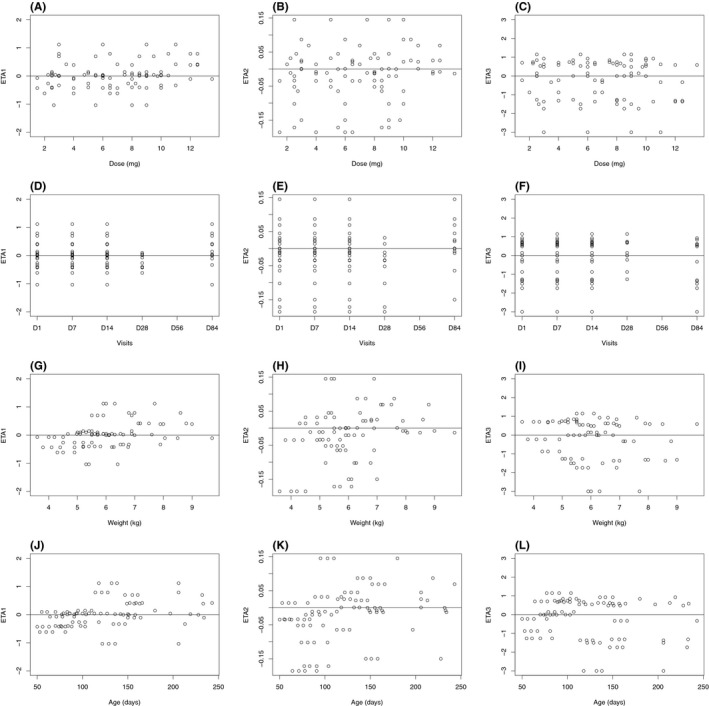
Propranolol base model: distributions of inter‐individual variabilities inter‐individual variability attached to apparent plasma clearance versus dose (A) visit number or time (D) weight (G) and age (J), inter‐individual variability attached to apparent volume of distribution versus dose (B) visit number or time (E) weight (H) and age (K), interindividual variability attached to first‐order absorption versus dose (C) visit number or time (F) weight (I) and age (L)

Diagnostic plots (Figure 2) demonstrated that population and individual predictions versus observations were equally distributed around the identity line. Most of WRES and CWRES were distributed between −2 and 2. No trend was observed on population or individual‐weighted residuals when plotted versus predicted concentration or time after dose. ETA distributions are given in Figure 3.

### 3.4 Covariate selection and final model

Only a weight effect was tested on the structural parameters. During forward selection, the inclusion of weight affecting the clearance resulted in a significant decrease in the OFV of 12 units (*P *< .001). The inclusion of weight effect on CL/F was performed by estimating the allometric exponent or by fixing the allometric exponent. AIC criteria was found lower using the allometric exponent fixed to 0.75 demonstrating that the exponent fixed to 0.75 is more appropriate.

Conversely, the inclusion of weight affecting volume of distribution did not alter the objective function. This result is consistent with the poor precision of the estimation of BSV on V (large CI including 0) and the large eta‐shrinkage (62.3%) reflecting the poor degree of information available to estimate the random effect independently. In this model the absence of weight effect just indicated that the available information did not support the identification of the classical weight effect on the volume of distribution.

The random effect model was refined: correlations between estimates of variance for BSV (ETA) were estimated between ETA(CL), ETA(V), and ETA(Ka) (block of 3 ETAs), between ETA(CL) and ETA(V), between ETA(CL) and ETA(Ka). No correlation was found to be significant. As explained above, the BSV on the volume of distribution remaining with a large CI including 0 was excluded from the final model.

The code for the final model is given in Equation 4 and the corresponding final estimates are given in Table 5.

(5) $$\begin{array}{cc} CL/F & =\theta_{1}∙{\left(\frac{\mathrm{Weight}(\mathrm{kg})}{6.3}\right)}^{0.75}∙\exp\left(\eta_{1}\right)\\ & V/F=\theta_{2}\\ & k_{a}=\theta_{3}∙\text{exp}\left(\eta_{2}\right) \end{array}$$

**Table 5:** Propranolol final model: parameter estimates

Description of effects		Symbol	Estimate	95% CI (RSE %)	CV%
Fixed effects	Allometric effect of Weight on CL/F: θ_1_ × (WGT/median weight)^0.75^				
	CL/F	θ_1_	19.3	15.4‐23.2 (10.3%)	
	V/F	θ_2_	122	104‐140 (7.32%)	
	Ka	θ_3_	0.993	0.558‐1.43 (22.4%)	
Random effects					
Between subjects’ variability	On CL/F	ω²_1_	0.195	0.0492‐0.341 (38.2%)	44.2
	On Ka	ω²_2_	1.75	0.370‐3.13 (40.2%)	132
Residual error	Proportional	σ²_1_	0.0953	0.0675‐0.123 (14.9%)	30.9

RSE, relative standard error of model parameter estimates obtained from the NONMEM covariance step; CI, confidence Interval calculated as estimate ± 1.96*RSE; CL/F, apparent total plasma clearance; V/F apparent volume of distribution; Ka: first‐order rate of absorption; θ; NONMEM fixed parameter; ω NONMEM between‐subject random effect; σ NONMEM residual random effect.

The value 6.3 is the median weight (in kg) observed in the total population from D1 to D84. The BSV on the clearance is 44.2% (eta‐shrinkage = 1%) and is higher on the absorption rate (BSV = 132%, eta‐shrinkage = 26%). For a typical patient of Group 1 of 5.6 kg (median weight in Group 1 at Day 28), the clearance is 17.2 L hour^−1^ (3.06 L hour^−1 ^kg^−1^). For a typical patient of Group 2 of 7.65 kg (median weight in Group 2 at Day 84), the clearance is 22.3 L hour^−1^ (2.92 L hour^−1^ kg^−1^).

The 95% CIs of each parameter did not contain zero. The RSE of fixed parameter estimates were <25% and <45% for random parameter estimates indicating a good and improved precision of estimates compared to the base model and no over parameterization of the model.^33^, ^34^

**Figure 4:**
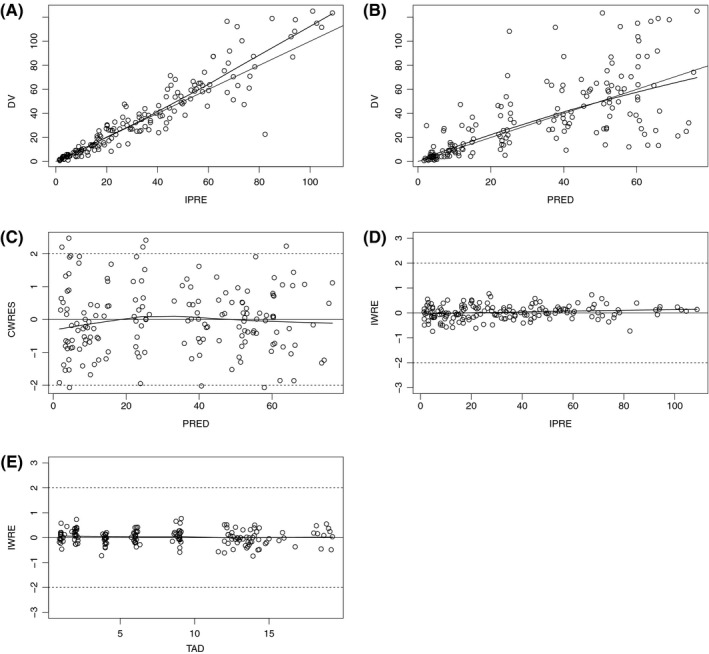
Final model goodness of fit plots Individual predictions versus observations (A) Population predictions versus observations (B) conditional population‐weighted residuals (CWRES) versus population predictions (C) Individual predictions versus time after dose (H) (D) and individual predictions versus individual‐weighted residuals (E)

**Figure 5:**
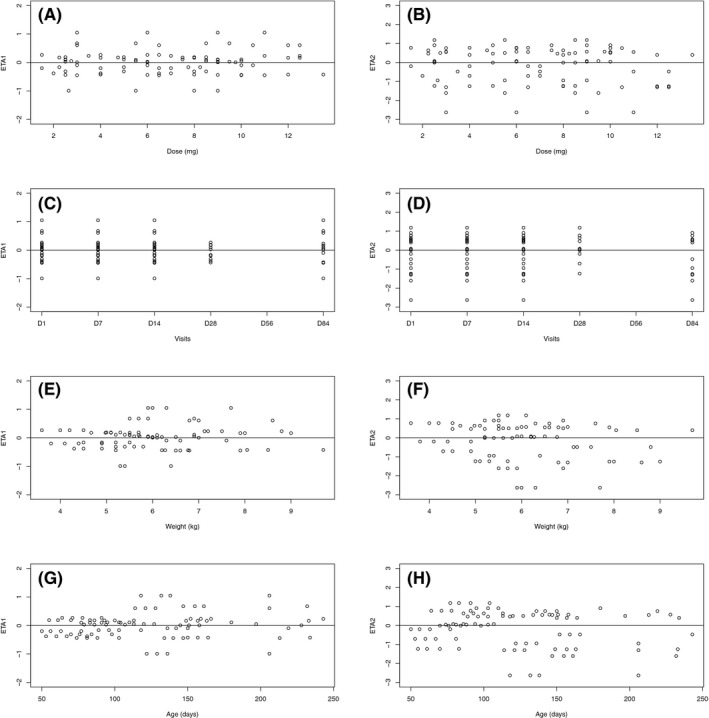
Propranolol final model: distributions of inter‐individual variabilities inter‐individual variability attached to apparent plasma clearance versus dose (A) visit number or time (C) weight (E) and age (G), interindividual variability attached to first‐order absorption versus dose (B) visit number or time (D) weight (F) and age (H)

As for the base model, population and individual predictions versus observations were equally distributed around the identity line; most of WRES and CWRES were distributed between −2 and 2. No trend was observed on population‐ or individual‐weighted residuals when plotted versus predicted concentration or time after dose (Figure 4). No trend was observed in clearance and ka ETA distributions (Figure 5).

### 3.5 Visual predictive check

**Figure 6:**
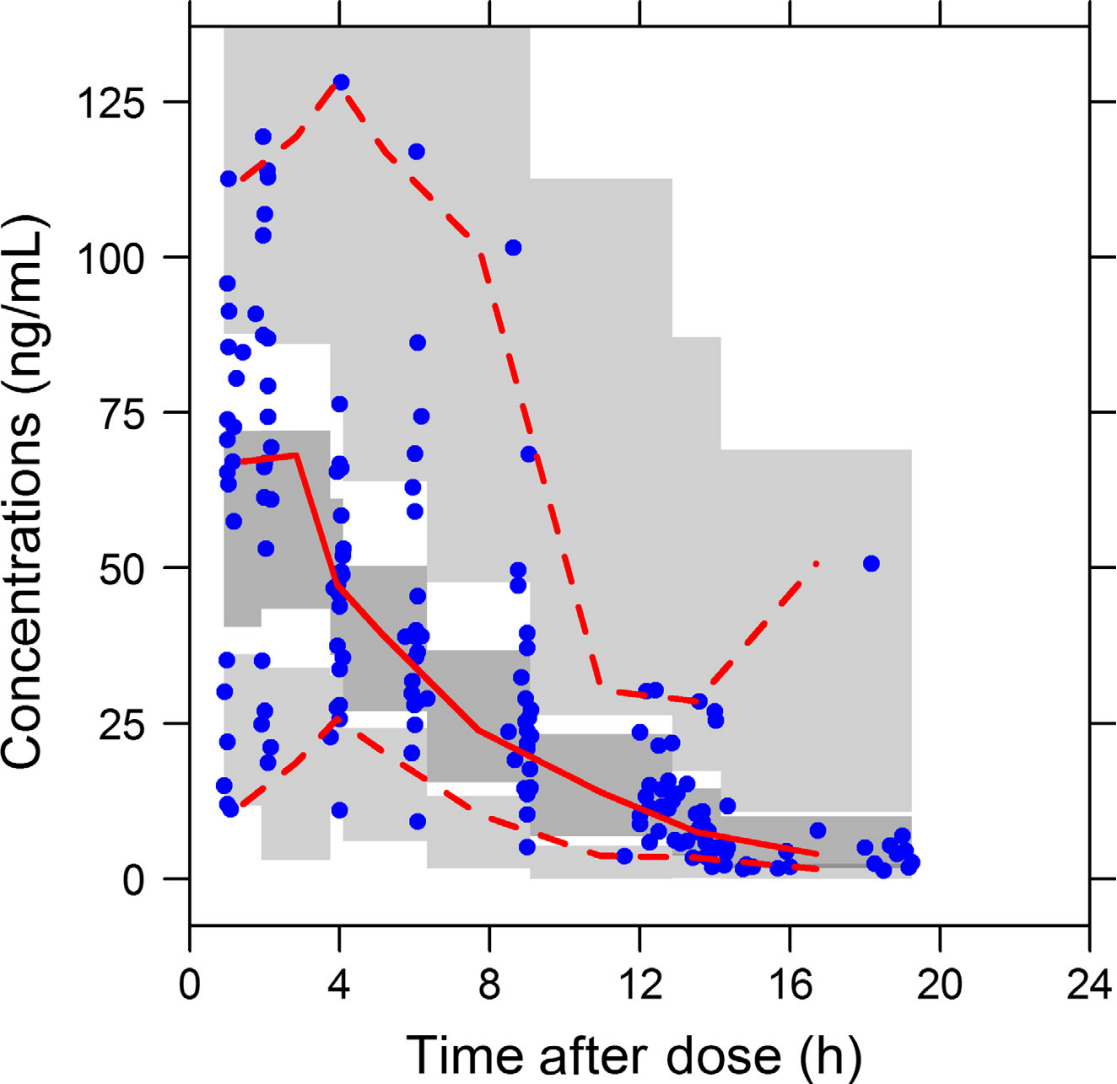
Pred‐corrected VPC for the final population pharmacokinetic model for oral propranolol comparing observed to simulated propranolol concentrations as a function of time. Dots correspond to pred‐corrected observations (propranolol concentrations divided by their respective population predictions); solid lines correspond to the 2.5%, 50%, and 97.5% of pred‐corrected observations. Dark gray and light gray areas correspond, respectively, to the 95% confidence interval of median and 2.5 and 97.5% of pred‐corrected simulations

The 5th, 50^th^, and 95th percentiles from the prediction corrected simulated data were superimposed with the observed concentrations in Figure 6 to visualize the predictive performance of the final PK model. Additional VPC performed by visit and coverage plots are given in Figures S1 and S2.

More than 90% of observed concentrations were included between the 5th and 95th percentiles of simulated concentrations. Based on the pcVPC and VPC results, the final model was judged to predict adequately the pharmacokinetics of propranolol in infants. Thus, simulations can be performed with confidence.

### 3.6 Simulations

**Figure 7:**
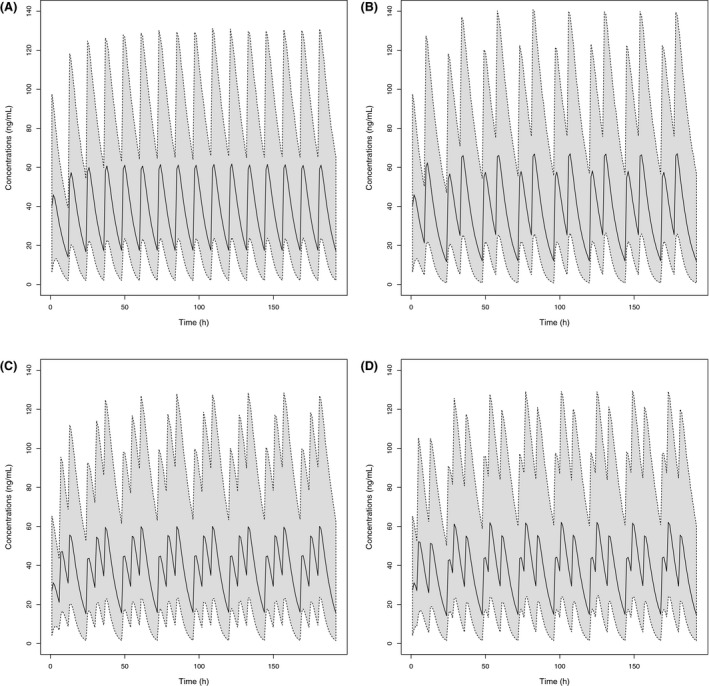
Simulation of different dosing regimens at 3 mg kg^−1^ day^−1^ of propranolol in infants. BID dosing regimen regular interval of 12 hour (A), BID dosing regimen with irregular dosing interval administrations at clock times 8:00 am and 5:00 pm over a 24 hour period (B), TID dosing regimen with administrations at clock times 8:00 am, 14:00 pm, and 8:00 pm (C), TID dosing regimen with at clock times 8:00 am, 12:00 am, and 8:00 pm (D). The solid line is the 50th percentile of simulated data and the dashed lines at the limits of gray area are the 5th and 95th percentiles of simulated data. BID, twice daily; TID, time daily dosing

Propranolol concentrations were predicted for four dosing regimens by Monte‐Carlo simulations (Figure 7).

Simulated median *C*_max_ were compared for the BID administration with regular 12‐hour dosing intervals and the alternative dosing regimens (Table 6): differences were <10% with the BID administration 9‐hour intervals and <5% for all the tested TID dosing regimens. Simulated median *C*_min_ showed a <20% differences between the regular 12‐hour BID regimen and all the tested alternative dosing regimens.

**Table 6:** 5th, 50^th^, and 95th percentiles of simulated *C*_min_ and *C*_max_ after repeated administration of 3 mg/kg/day of propranolol in infants

Dosing regimen	*C*_min_ (ng/mL)	*C*_max_ (ng/mL)
Regular 12 hour interval	1.99, 14.0, 39.4	24.0, 61.8,131
Administration at clock times 8:00 am and 5:00 pm	0.803, 11.6, 47.0	26.4, 67.1,141
Administration at clock times 8:00 am, 2:00 pm, and 8:00 pm	1.43, 14.2, 50.2	24.7, 62.2, 130
Administration at clock times 8:00 am, 12:00 am and 8:00 pm	1.53, 15.2, 43.4	23.6, 60.1, 128

## 4 DISCUSSION

The main objectives of this analysis were to describe the pharmacokinetics of propranolol in infants, to evaluate the BSV and to understand the source of this BSV.

The analysis was performed using 167 concentrations data from 22 patients (6 males and 16 females) who were sampled twice during the titration period (1‐2 mg kg^−1^ day^−1^) and 6 times during the target dose period (3 mg kg^−1^ day^−1^). The treatment was given BID.

A one‐compartment model with a first‐order absorption rate and a first‐order elimination rate was the most appropriate model to describe the data. The BSV on the clearance, whatever the group of age, was around 40 %, which is close to that described in the literature for adult exposure.^21^ With a limited number of blood samples, sampling collection was not designed to accurately estimate the absorption rate and the volume of distribution. Consequently, BSV on the absorption rate and the corresponding RSE were high (132% and 40.2%, respectively). However, the corresponding eta‐shrinkage remained acceptable (26%). On the contrary, no BSV was estimated on the volume: taken together the absence of improvement of the Fobj when the BSV was added, the large CI of the BSV including 0 and the large eta‐shrinkage on V/F reflecting the poor degree of information available to estimate the random effect independently, it was deemed that the inclusion of BSV on V was not useful in the model. The residual error which represents a composite of assay and intrasubject variabilities, model misspecification, errors in the timing of dose administration or sample collection, subject noncompliance, and other unexplained errors, was estimated at 30.9%.^30^ This residual variability was considered reasonable with ambulatory infant patients.

The weight was found to impact the clearance according to an allometric function whereas no weight effect was evidenced to affect the volume of distribution. The absence of identified weight effect on V/F is consistent with the poor precision of the estimation and the large eta‐shrinkage of BSV on V. In this model the absence of weight effect on V/F just indicated that the current information available did not support the identification of the classical and physiological weight effect on the volume of distribution. The allometric component on CL/F was fixed to 0.75 as the AIC criteria demonstrated that the exponent fixed to 0.75 (instead of estimated) was more appropriate. It is widely described that body size is the primary covariate to investigate in the pediatric population and allometric scaling using an empiric power exponent of 3/4 is superior to other techniques (ie, using body surface area),^3^, ^4^, ^5^, ^37^ Moreover it is recognized that a wide imprecision of empirical estimates of allometric exponents is obtained when estimated from typical sized datasets with limited numbers of subjects and distribution of weights means.^17^ Finally, it was deemed that fixing the allometric exponent was an acceptable methodology.

Although it is recognized that a maturation model should be added in population PK models in infants to take into account the growth and the maturation of elimination/metabolism processes, one can conclude that for this specific model of propranolol in infants, the influence of ontogeny on the structural parameters was minimal for the considered period of age evaluated in this analysis. This could be explained by the characteristics of propranolol transformation which is balanced between several elimination pathways whose maturation rates are different.^2^, ^38^, ^39^

Geometric means of individual predicted clearances were 16.3 L hour^−1^ and 24.3 L hour^−1^ (Table 7) for Group 1 and Group 2, respectively. When compared to the geometric means of observed clearances (15.2 L hour^−1^ and 25.5 L hour^−1^, respectively) calculated by noncompartmental analysis (Del Frari Poster at SPD2015^10^; both predicted and observed values were close supporting the good adequation of the model to the observed data. This emphasizes that the simple covariate model on the apparent plasma clearance (influence of weight only) is suitable to describe adequately the clearance and its associated variability.

**Table 7:** Individual values and descriptive statistics of apparent clearance of propranolol predicted using the final model

Group	Visit	Patient ID	CL/F (L/h)
1	Day 28	50105	20.7
		50106	21.5
		50109	10.9
		50110	13.1
		50201	14.8
		50204	13.2
		50302	20.2
		50303	19.5
		50305	13.2
		50306	20.7
N			10
Geometric mean (CV%)			16.3 (25.8%)
Median (range)			17.2 (10.9‐21.5)
2	Day 84	50101	64.1
		50103	21.7
		50104	29.8
		50107	31.2
		50108	14.7
		50202	7.19
		50203	21.0
		50205	44.6
		50301	23.0
		50304	20.0
		50401	17.5
		50402	40.3
N			12
Geometric Mean (CV%)			24.3 (62.1%)
Median (range)			22.3 (7.19‐64.1)

ID, Identification, CL/F, apparent total plasma clearance.

Finally, the population PK model showed that the dose in mg/kg should be used without dose adaptation by range of age.

For a typical patient of 6 kg, total apparent clearance is 3.1 L hour^−1^ kg^−1^. This value is higher than those observed in Filippi (2013)^14^ which are between 1.47 and 1.75 L hour^−1^ kg^−1^. These data could not be fully compared because the evaluation was performed mainly in preterm neonates (32 preterm and 4 term newborns) whereas the present evaluation was performed in infants (50‐151 days at inclusion).

These values of clearance are in the same range of data reported in adults after oral propranolol administration: 5.2‐3 L hour^−1^ kg^−1^ for single doses from 10 to 40 mg, respectively,^26^ 2.4 and 3.9 L hour^−1^ kg^−1^ after single dose of 80 mg in women and in men, respectively,^38^ 2.16 L hour^−1^ kg^−1^ after repeated TID administration of 80 mg.^31^

On the basis of the GOF plots and pcVPCs, the pharmacokinetic model was shown to describe and predict accurately the pharmacokinetics of propranolol in infants after repeated oral administration of 3 L hour^−1^ kg^−1^. An external evaluation would have been necessary to investigate the reliability and the performance of this model. Unfortunately, no blood sampling for propranolol determination were done in the phase 2/3 dose ranging study,^23^ and the data obtained in Filippi (2013) were from a different infant population (mainly preterm neonates enrolled to evaluate the safety and efficacy of propranolol in the retinopathy of prematurity) and thus not appropriate for the external qualification.

The phase 2/3 dose ranging study has demonstrated the efficacy and tolerability of the new pediatric solution of propranolol at the dose of 3 mg kg^−1^ day^−1^ given BID. However, the available literature on the treatment of IH largely documented TID dosing regimen.^11^, ^13^, ^16^, ^42^ Considering that repeated administration of treatment in infants could not always be a regular dosing regimen (due to possible irregular sleeping periods and meal times), irregular BID dosing regimen and 2 different TID dosing regimens were simulated and compared with the regular BID dosing regimen investigated in the clinical PK study. Whatever the dosing regimen, simulations confirmed that infants are exposed to propranolol over a 24 hour‐period. Simulated median *C*_min_ and *C*_max_ exhibit a <20% difference compared to the regular BID regimen.

With regard to safety, cardiovascular adverse events being known to be related with peak concentrations, the similarity between simulated *C*_max_ after BID or TID daily dosing clears the risk of majored cardiovascular adverse events after BID dosing compared to TID dosing. This has been confirmed during the phase 2/3 clinical study.

Finally, the efficacy and safety being demonstrated in the clinical phase 2/3 in infants after a BID regimen, and simulations having shown similar minimum and maximum concentrations between BID and TID regimen, one can consider that the BID regimen is adequate to maintain efficacy with a good tolerability. The simulations having shown that an irregular BID dosing regimen in terms of time interval between the 2 administrations had a limited impact on median *C*_max_ and *C*_min_ values, an early administration in the evening is possible allowing to avoid sleep disturbances which are known adverse events with propranolol.

To conclude, a population pharmacokinetic model using an allometric weight function on apparent clearance was established and adequately predicted the pharmacokinetics of propranolol in infants after repeated oral BID administrations of 3 mg kg^−1 ^day^−1^ of propranolol. This analysis confirmed that the dose of propranolol prescribed in mg/kg should be used without dose adaptation by range of age.

The PK model was used to simulate concentrations after four different dosing regimen: twice daily versus three times daily and strict 12 hour‐interval dosing regimen versus irregular twice daily dosing intervals. The simulations showed similar minimum and maximum concentrations suggesting that no majoration of cardiovascular events are anticipated with a BID regimen. These results support the use of a twice daily dosing preferably to a three times daily dosing. In addition, simulations showed the possibility of irregular twice daily dosing which allow early administration when needed.

## DISCLOSURE

C. Léauté‐Labrèze has received fees as expert and consultant for Pierre Fabre Dermatologie, she is one of the inventors involved in the patent for the use of a beta blocker for the manufacture of a medicament for the treatment of hemangiomas. S. Barbarot has received research grants from Pierre Fabre Laboratory and Fondation pour la Dermatite Atopique. C. Chaumont, A. Delarue, and J.J. Voisard are full time employees of Pierre Fabre Dermatologie. L. Del Frari is a full time employee of drug company Pierre Fabre Médicament. V. Brunner is full time employee at SERVIER Laboratories and is a former employee at Pierre Fabre Médicament. Pierre Fabre Dermatologie provided financial and material support for the design and concept of the study from which the data used for modeling and simulation analysis are come from, data collection, data management, data analysis, and the publication of this article. J.P. Lacour and L. Guibaud have no conflicts of interests to declare.
