# Supplementary material for: Propranolol pharmacokinetics in infants treated for Infantile Hemangiomas requiring systemic therapy: Modeling and dosing regimen recommendations
Source: Pharmacol Res Perspect. 2018 Apr 30;6(3):e00399. doi: 10.1002/prp2.399 (PMC5925426; doi:10.1002/prp2.399)
Supplement: Supplementary file 1 [file PRP2-6-e00399-s001.docx]

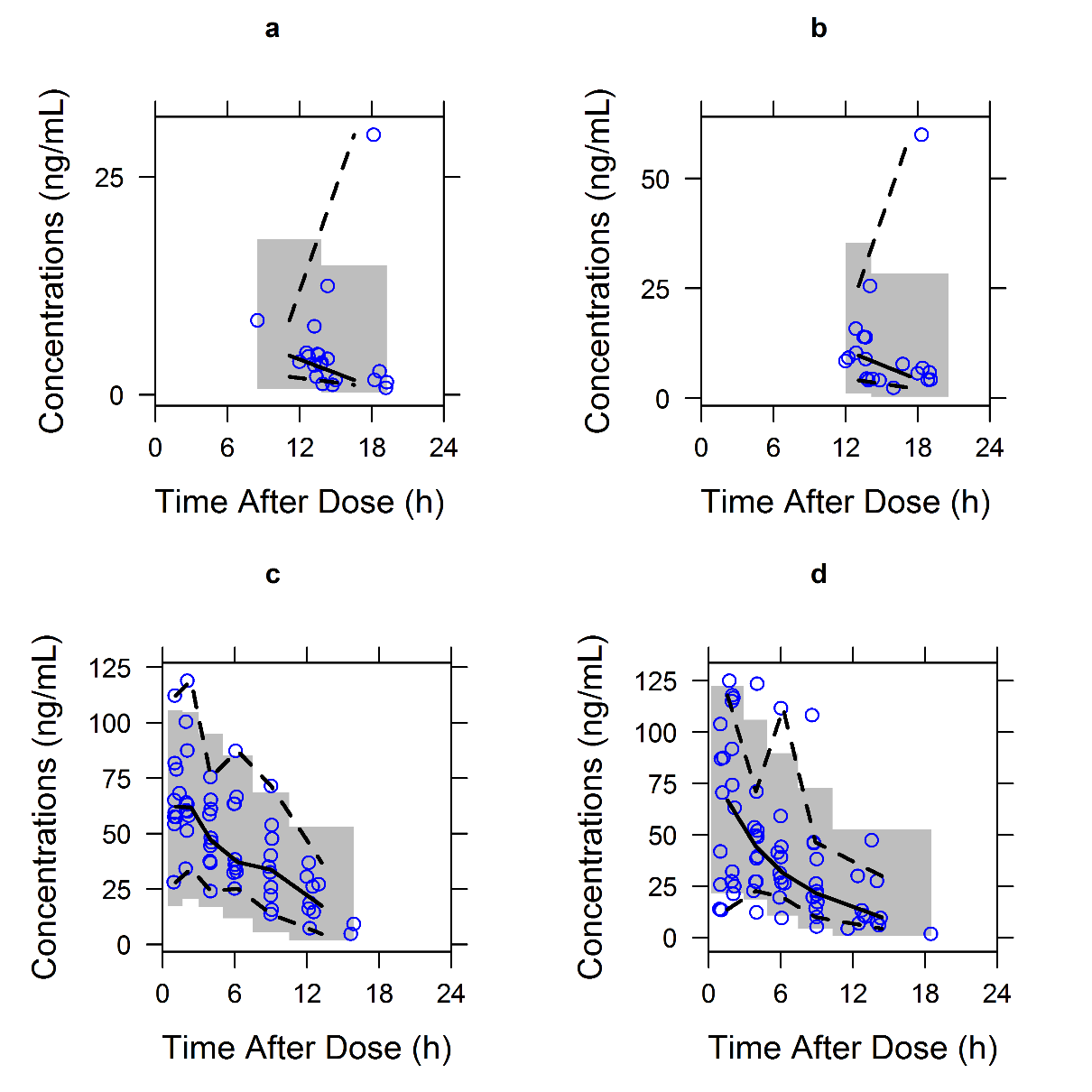


Figure S1 Visual Predictive Check for the final population pharmacokinetic model for oral propranolol by visit: on Day 7 (a) and Day 14 (b), on Day 28 (c) and on Day 84 (d). The solid line is the 50^th^ percentile of observed data, the dashed lines are the 5^th^ and 95^th^ of observed data, the limits of grey area are the 5^th^ and 95^th^ percentiles of simulated data. The circles represent observed data.


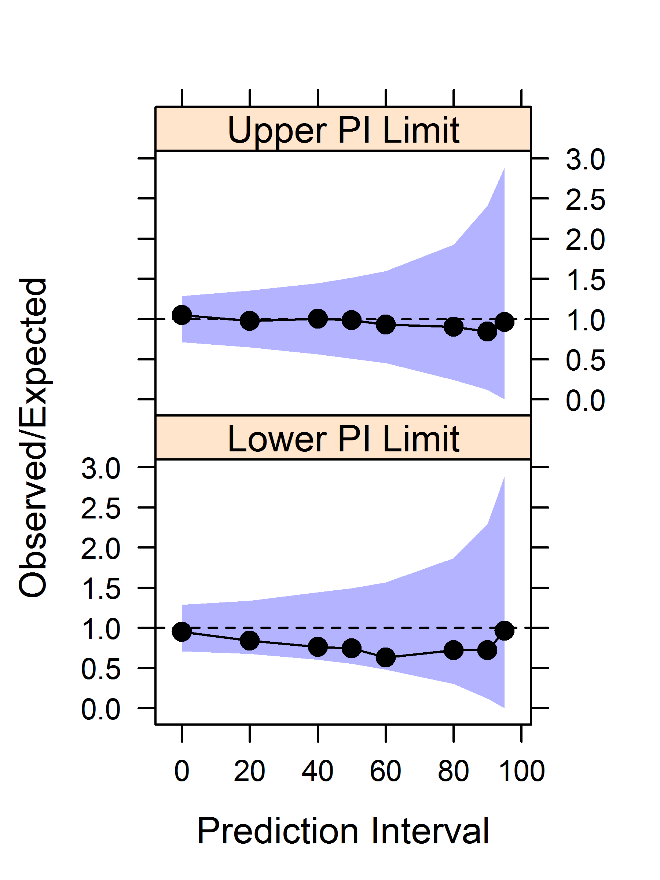


Figure S2 Coverage plots at different prediction intervals for the final population pharmacokinetic model for oral propranolol. The plot shows the relative amount of data points outside of their PI compared to the expected amount at that PI. In addition a confidence interval around these values are computed based on the simulated data.
